# Nintedanib and Dasatinib Treatments Induce Protective Autophagy as a Potential Resistance Mechanism in MPM Cells

**DOI:** 10.3389/fcell.2022.852812

**Published:** 2022-03-22

**Authors:** Luca Hegedüs, Kata D. Szücs, Matthias Kudla, Julian Heidenreich, Verena Jendrossek, Samuel Peña-Llopis, Tamas Garay, Andras Czirok, Clemens Aigner, Till Plönes, Silvia Vega-Rubin-de-Celis, Balazs Hegedüs

**Affiliations:** ^1^ Department of Thoracic Surgery, University Medicine Essen - Ruhrlandklinik, West German Cancer Center, University of Duisburg-Essen, Essen, Germany; ^2^ Institute of Cell Biology (Cancer Research), Essen University Hospital, Essen, Germany; ^3^ Translational Genomics in Solid Tumors, German Cancer Consortium (DKTK) and German Cancer Research Center (DKFZ) at the University Hospital Essen, Essen, Germany; ^4^ Division of Solid Tumor Translational Oncology, German Cancer Consortium (DKTK) and German Cancer Research Center (DKFZ), Heidelberg, Germany; ^5^ Faculty of Information Technology and Bionics, Pazmany Peter Catholic University, Budapest, Hungary; ^6^ Division of Oncology, Department of Internal Medicine and Oncology, Semmelweis University, Budapest, Hungary; ^7^ Department of Biological Physics, Eötvös University, Budapest, Hungary

**Keywords:** malignant pleural mesothelioma, autophagy, TFEB, nintedanib, dasatinib

## Abstract

Malignant pleural mesothelioma (MPM) is a rare type of cancer with a grim prognosis. So far, no targetable oncogenic mutation was identified in MPM and biomarkers with predictive value toward drug sensitivity or resistance are also lacking. Nintedanib (BIBF1120) is a small-molecule tyrosine kinase inhibitor that showed promising efficacy preclinically and in phase II trial in MPM as an angiogenesis inhibitor combined with chemotherapy. However, the extended phase III trial failed. In this study, we investigated the effect of nintedanib on one of its targets, the SRC kinase, in two commercial and six novel MPM cell lines. Surprisingly, nintedanib treatment did not inhibit SRC activation in MPM cells and even increased phosphorylation of SRC in several cell lines. Combination treatment with the SRC inhibitor dasatinib could reverse this effect in all cell lines, however, the cellular response was dependent on the drug sensitivity of the cells. In 2 cell lines, with high sensitivity to both nintedanib and dasatinib, the drug combination had no synergistic effect but cell death was initiated. In 2 cell lines insensitive to nintedanib combination treatment reduced cell viability synergisticaly without cell death. In contrast, in these cells both treatments increased the autophagic flux assessed by degradation of the autophagy substrate p62 and increased presence of LC3B-II, increased number of GFP-LC3 puncta and decreased readings of the HiBiT-LC3 reporter. Additionaly, autophagy was synergistically promoted by the combined treatment. At the transcriptional level, analysis of lysosomal biogenesis regulator Transcription Factor EB (TFEB) showed that in all cell lines treated with nintedanib and to a lesser extent, with dasatinib, it became dephosphorylated and accumulated in the nucleus. Interestingly, the expression of certain known TFEB target genes implicated in autophagy or lysosomal biogenesis were significantly modified only in 1 cell line. Finally, we showed that autophagy induction in our MPM cell lines panel by nintedanib and dasatinib is independent of the AKT/mTOR and the ERK pathways. Our study reveals that autophagy can serve as a cytoprotective mechanism following nintedanib or dasatinib treatments in MPM cells.

## Introduction

Malignant pleural mesothelioma (MPM) is a rare type of cancer that originates from the mesothelial cells of the pleura. Its occurrence is strongly associated with asbestos exposure following a 20 to 30 years-long latency period. The median overall survival of MPM patients is around 8–14 months and currently no targeted therapy is approved as a treatment for this type of cancer. This can be explained by the fact that oncogenic mutations are rarely present in MPM tumors and the most frequently mutated genes are tumor suppressors such as *BAP1*, *NF2* and *CDKN2A* (Nicolini et al., 2019). Accordingly, there is no established molecular classification of MPM that could serve as a predictive marker for current therapeutic options.

Macroautophagy (here thereafter referred to as autophagy) is a degradation pathway whereby intracellular components are engulfed into double membraned structures (autophagosomes) for their degradation into the lysosome. Autophagy plays an essential role in both health and disease and it is a highly conserved pathway across eukaryotes. It is essential in maintaining cellular homeostasis to provide energy and to eliminate damaged organelles, protein aggregates or pathogens. Autophagy has also been involved in multiple human pathologies, including cancer (Levine and Kroemer, 2019). It is becoming clear that autophagy plays a dual role in cancer, and whereas it has a protective function against tumorigenic insults at the early stages of tumor development, it also has a pro-tumorigenic role in established tumors (Vega-Rubin-de-Celis, 2021).

Autophagy is regulated at multiple levels. For instance, the so-called autophagy-related (*ATG*) genes are evolutionary conserved and regulate multiple steps in the pathway, some of them controlling autophagosome formation and both selective and non-selective autophagy (Vega-Rubin-de-Celis, 2021). Autophagy is initiated upon multiple signals, including nutrient and stress clues that are mediated by mTORC1 (mechanistic target of Rapamycin complex 1) and AMPK (AMP activated protein kinase), or by autophagy cargoes from multiple sources such as damaged organelles or unfolded proteins that are mediated by ATG11. Active mTORC1 phosphorylates and inactivates ULK1 (UNC-51-like kinase 1), the protein kinase unit of the initiation complex, that also contains ATG13, ATG11 and ATG101. Upon autophagy-inducing conditions, this initiation complex translocates to the ER and initiates autophagosome formation. AKT1 (Protein kinase B) is an upstream regulator of mTORC1 that modulates its activity by phosphorylating and inactivating the TSC1/TSC2 complex as well as by direct phosphorylation of the mTORC1 component PRAS40 (Proline-rich AKT substrate 40), leading to mTORC1 activation and autophagy inhibition (Laplante and Sabatini, 2012). An mTORC1-independent autophagy regulation function of AKT1 was also described through direct phosphorylation of Beclin 1 (Wang et al., 2012), and through regulation of XIAP (X-linked inhibitor of apoptosis), inhibiting basal levels of autophagy (Huang et al., 2013). In low-energy conditions, AMPK phosphorylates ULK1, activating the complex. Once activated, ULK1 phosphorylates components of the PI3KC3 (class III phosphatidylinositol 3-kinase) complex that includes ATG14, Beclin 1, VPS34 and VPS15, generating PI3P on the autophagosome-initiating membranes. mTORC1 and AKT also regulate autophagy and lysosomal biogenesis at the transcriptional level through modulating the localization of the transcription factor EB (TFEB), a master regulator of lysosomal biogenesis (Sardiello et al., 2009) and some autophagy genes (Settembre et al., 2011). TFEB is regulated by mTORC1 (Peña-Llopis et al., 2011), and phosphorylation by mTORC1 at residues S122 (Vega-Rubin-de-Celis et al., 2017), S142 (Settembre et al., 2012; Nezich et al., 2015) and S211 (Martina et al., 2012; Roczniak-Ferguson et al., 2012; Settembre et al., 2012; Vega-Rubin-de-Celis et al., 2017) has been shown. In addition, AKT phosphorylates TFEB at S467 independently of mTORC1 to promote its cytosolic retention (Palmieri et al., 2017). TFEB is also phosphorylated by ERK1/2 at S142 and this modification retains TFEB in the cytosol (Settembre et al., 2011).

A number of studies have shown that autophagy plays an important role in the regulation of cell death and therapy resistance in MPM tumors. Autophagy induction might contribute to the malignant transformation upon asbestos exposure, when DNA damage and inflammation are induced through the release of the high mobility group box 1 (HMGB1) protein by mesothelial cells and macrophages. HMGB1 also initiates autophagy by interacting with RAGE receptor regulating the mTORC1-ULK1 pathway, and through Beclin one phosphorylation, promoting the survival of the transformed mesothelial cells (Xue et al., 2020). It was shown that both mesothelioma cell lines and tumor spheroids can strongly differ in their basal autophagy level and this influences their sensitivity to therapy. Both PI3K/mTOR dual inhibitors and BCL-XL-selective BH3 mimetic treatments induced protective autophagy in MPM cells, although combination with autophagy inhibitors could initiate cell death (Echeverry et al., 2015; Xu et al., 2021). Furthermore, in MPM tumor spheroids treatment with an inhibitor of autophagy initiation through ULK1 inhibition, MRT68921, increased chemosensitivity in spheroids with high autophagy steady-state level (Follo et al., 2018).

Nintedanib (BIBF1120) is a small-molecule tyrosine kinase inhibitor (TKI) that inhibits the activity of several receptor tyrosine kinases (TKs), such as vascular endothelial growth factor receptor (VEGFR 1–3), platelet-derived growth factor receptor (PDGFR-α and–β) and fibroblast growth factor receptor (FGFR 1–3). It also impairs the activity of certain non-receptor TKs including the SRC-kinase family member Lyn (tyrosine-protein kinase lyn), Lck (lymphocyte-specific tyrosine-protein kinase) and SRC (proto-oncogene tyrosine-protein kinase src). It binds to the ATP-binding pocket of these receptors in a competitive and reversible manner (Wind et al., 2019). Nintedanib was approved by the FDA for the treatment of idiopathic pulmonary fibrosis (IPF), a chronic lung disease, as it effectively interferes with lung fibroblast proliferation and migration (Rivera-Ortega et al., 2018). It is also used for the treatment of locally advanced, metastatic or recurrent non-small cell lung carcinoma in combination with docetaxel due to its antiangiogenic effect (Reck et al., 2014). The effect of nintedanib on MPM tumors has also been investigated. In preclinical experiments, nintedanib inhibited the proliferation and migration of MPM cells *in vitro* and decreased tumor burden and vascularization *in vivo* (Laszlo et al., 2018). In the first clinical study (LUME-Meso phase II/III trial), combination of nintedanib with standard-of-care chemotherapy provided a clinical benefit (Grosso et al., 2017). Unfortunately, the extended phase III trial did not confirm this initial observation (Scagliotti et al., 2019). These results highlight the need to further examine the effect of nintedanib in MPM cells in order to identify potential biomarkers of sensitive tumors and to explore possible resistance mechanisms. It was demonstrated that in lung cancer cells with hyperactivated FGFRs nintedanib can be sequestered into the lysosome as an intrinsic resistance mechanism. In the lysosome, the weakly basic nintedanib becomes protonated and trapped. Upon co-treatment with the lysosomal vacuolar H + -ATPase (V-ATPase) inhibitor bafilomycin A1 or chloroquine, the lysosomal accumulation of nintedanib decreased as lysosomal acidification was inhibited. Furthermore, both drugs sensitized the cells to nintedanib already at subtoxic doses (Englinger et al., 2017). In lung fibroblasts from patients with IPF nintedanib initiated autophagy in a Beclin 1-dependent and ATG7-independent manner (Rangarajan et al., 2016).

Dasatinib is an inhibitor of primarily the SRC family kinases (SRC, LCK, YES, FYN) and BCR-ABL, but it has an effect also on other receptor TKs, including c-KIT, PDGFR-α and PDGFR-β. It is approved for chronic myeloid leukemia and Philadelphia chromosome positive acute lymphoblastic leukemia (Lindauer and Hochhaus, 2014). c-SRC is highly expressed and activated in both MPM cell lines and MPM tumor samples, and dasatinib treatment induces cell cycle arrest and inhibits the migration and invasion of MPM cells (Tsao et al., 2007). Clinically, as a neoadjuvant agent, dasatinib did not show significant efficacy among patients with resectable MPM, even though, reduced SRC phosphorylation at Tyr419 after dasatinib treatment correlated with longer progression-free survival (PFS) (Tsao et al., 2018). Also, as a second line treatment after chemotherapy in patients with unresectable MPM it had limited efficacy (Dudek et al., 2012). Similarly to nintedanib, it was found that dasatinib can induce autophagy in some cancer cells. In ovarian cancer cell lines, dasatinib initiated autophagic cell death and decreased cell growth in an AKT, mTOR and Beclin 1-dependent manner (Le et al., 2010). In another study where the autophagy-inducing capacity of different TKIs was compared, dasatinib initiated autophagy in two lung cancer cell lines but not in an oral squamous cell carcinoma cell line and in this case, autophagy had a cytoprotective effect (Tanaka et al., 2020).

In this study we investigated the combined effect of dasatinib and nintedanib treatments on a panel of mesothelioma cell lines. Surprisingly, we found that nintedanib treatment did not inhibit SRC activation in MPM cells and paradoxically even increased phosphorylation of SRC in several cell lines. Combination treatment with SRC inhibitor dasatinib could reverse this effect in all cell lines but its impact on cell viability was cell line-dependent. In some cell lines the treatments initiated cell death while in others induced protective autophagy to promote resistance.

## Materials and Methods

### Cell Lines and Compounds

SPC111 cell line was purchased from Sigma-Aldrich (St. Louis, MO, United States). SPC212 cell line was a kind gift from Prof. R. Stahel (University of Zurich, Zurich, Switzerland). Both cell lines were established from human MPM tumors with biphasic morphology (Stahel et al., 1988). Novel cell lines PF626, PF142, PF531, PF434, PF588 and PF655 were established by our group from the pleural effusion sample of MPM patients (Table 1). The pleural fluid was centrifuged at 1,200 × *g* for 10 min and the supernatant was aliquoted for further use. A cell culture was initiated from the pellet in RPMI1640 media containing 10% FBS, and 100 U/ml penicillin-streptomycin in a 25 cm^2^ tissue culture flask. Cells were passaged at least 8 times in order to eliminate the non-malignant cells prior to the experiments. The Ethics Committee at the University Hospital Essen (#18-8208-BO) approved the study and the patients provided informed consent. Cell lines were subjected to Single Nucleotide Analysis by Multiplex Cell Line Authentication (Multiplexion, Heidelberg, Germany). All cell lines were further cultured in DMEM media supplemented with 10% FBS and 1% penicillin-streptomycin at 37°C in a humidified atmosphere with 5% CO_2_.

**TABLE 1:** Patient characteristics of the newly established MPM cell lines. PF588 and PF655 cell lines (in bold) are derived from the same patient.

Cell line	Age	Gender	Asbestos	Disease course	Histology
PF 142	64	female	yes	diagnostic	biphasic
PF 434	86	male	yes	diagnostic	sarcomatoid
PF 531	57	male	yes	diagnostic	sarcomatoid
**PF 588**	64	female	yes	diagnostic	epithelioid
PF 626	74	male	yes	diagnostic	epithelioid
**PF 655**	65	female	yes	post-chemo	epithelioid

Dasatinib was purchased from Selleck Chemicals (Houston, TX, United States). It was dissolved in DMSO at 10 mM and stored at -80°C. Nintedanib (BIBF1122) was provided by Boehringer Ingelheim (Germany), it was dissolved in DMSO at 100 mM and kept at -20°C. Bafilomycin A1 was from MedChem Express. It was dissolved in DMSO to 1 mM and stored at -20°C. 3-Methyladenine (3-MA) was purchased from Selleck Chemicals (Houston, TX, United States) and it was dissolved in DMEM at 10 mM concentration.

### Viability Assay

We used Sulforhodamine B (SRB) assay to measure cell viability as described previously (Garay et al., 2015). Briefly, 3000 to 6000 cells were plated on the inner wells of 96-well plates and incubated for 24 h. Then, the cells were treated with increasing concentrations of dasatinib (25 nM, 50 nM, 0.1, 0.4, 1 μM) or nintedanib (0.3, 1, 3, 5 μM) for 72 h. For combination treatment, cells were treated with the different concentrations of dasatinib (25 nM, 50 nM, 0.1 μM, 0.4 μM) and nintedanib (1 μM, 3 μM, 5 μM) in all combinations. Then we washed the cells once with PBS and fixed them by adding ice-cold 6% trichloroacetic acid (TCA) to each well. After 1-h incubation at 4°C the plate was washed with distilled water and dried for 1–2 days. Then we stained the plate with 0.4% SRB dye (Sigma-Aldrich, St. Louis, MO, United States) for 15 min. Excess dye was removed by washing the plate with 1% acetic acid several times. After drying the plate for 1–2 h 10 mM Tris buffer was added to dissolve the protein-bound dye. Optical density was read at 570 nm with a microplate reader (EL800, BioTec Instruments, Winooski, VT, United States). Half-maximal inhibitory concentration (IC50) and combination index (CI) were calculated with CompuSyn software (ComboSyn Inc., Paramus, NJ, United States). Calculation of the CI values was based on the average of the viability values of three independent measurements. CI values indicates synergism (CI < 0.9), additive effect (CI is between 0.9 and 1.1) or antagonism (CI > 1.1).

### Cell Cycle Analysis

Cells were seeded on 6-well plates (1.5–3x10^5^ cells/well) and incubated over night. Then, we applied dasatinib (50 nM, 300 nM) and nintedanib (1 μM) treatments alone and in combinations for 72 h. After treatment both, the floating and adherent cells, were collected, centrifuged and washed once with PBS. After subsequent centrifugations, the cells were treated for 5 min with lysis buffer (Solution 10, 910-3010, Chemometec, Denmark) supplemented with the DAPI staining solution (Solution 12, 910-3012, Chemometec). The reaction was stopped with the stabilization buffer (Solution 11, 910-3011, Chemometec) and fluorescence was measured with NucleoCounter NC-3000system (Chemometec). Based on the amount of the fluorescent nuclear stain (DAPI) the DNA content of the cells could be determined.

### Western Blot Analysis

Cells were seeded on 6-well plates (1.5–3x10^5^ cells/well) and incubated over night. Then dasatinib (50 nM, 300 nM) and nintedanib (1 μM) treatments alone and in combinations were added for 24 h. To isolate proteins, cells were washed twice with PBS and 1 ml ice-cold 6% TCA solution/well was added to precipitate cellular protein. After incubation 1–24 h at 4°C the precipitate was collected and centrifuged for 10 min at 4°C. Subsequently, the pellets were resuspended in electrophoresis sample buffer (62.5 mM Tris–HCl, pH 6.8, 2% SDS, 10% glycerol, 5 mM EDTA, 125 mg/ml urea, 100 mM dithiothreitol). 20–30 μg of total protein was loaded on 10% SDS polyacrylamide gels. The primary antibodies used for the experiments are listed in Table 2. For detection HRP-conjugated anti-rabbit and anti-mouse secondary antibodies (Jackson ImmunoResearch, dilution 1:10,000) were used and luminography was performed with Pierce ECL Western Blotting Substrate (Thermo Scientific, Waltham, MS, United States).

**TABLE 2:** List of the primary antibodies used for western blot analysis.

Name	Catalog #	Company	Dilution
Phospho-Akt (Ser473) (193H12)	4058	Cell Signaling	1:1,000
Akt	9272	Cell Signaling	1:1,000
Phospho-p44/42 MAPK (Erk1/2) (Thr202/Tyr204) (D13.14.4E) XP	4370	Cell Signaling	1:1,000
p44/42 MAPK (Erk1/2) (L34F12)	4696	Cell Signaling	1:1,000
Phospho-FAK (Tyr576/577)	3281	Cell Signaling	1:1,000
Phospho-FAK (Tyr397) (D20B1)	8556	Cell Signaling	1:1,000
FAK	3285	Cell Signaling	1:1,000
Phospho-Src Family (Tyr416)	2101	Cell Signaling	1:1,000
Src (32G6)	2123	Cell Signaling	1:1,000
Phospho-S6 Ribosomal Protein (Ser240/244)	2215	Cell Signaling	1:1,000
β-Actin (13E5)	4970	Cell Signaling	1:1,000
SQSTM1 (p62) (D5L7G)	88,588	Cell Signaling	1:1,000
β-Actin-HRP	sc-47778	Santa Cruz	1,5000
Tubulin	T6074	Sigma	1,5000
LC3B	NB100-2220	Novus Biologicals	1:1,000
TFEB	A303-673A	Bethyl	1:1,000
Menin	A300-105A	Bethyl	1:1,000

### Plasmids

pBabe-GFP-LC3 was a generous gift from Prof. N. Mizushima (University of Tokyo) through Dr. Beth Levine (University of Texas Southwestern Medical Center). Autophagy LC3-HiBiT reporter vector was from Promega.

### Transfection

Plasmid transfection of cells were performed with the Mirus-LT1 transfection reagent according to the manufacturer’s instructions (Mirus Bio).

### Generation of Stable Cell Lines and HiBiT-LC3 Reporter Measurements

To generate cells stably expressing the LC3-HiBiT reporter, cells were transfected with the corresponding plasmid. Two days later cells were selected by incubation with media containing 250 μg/ml G418 (Gibco).

Analysis of the effects of treatments on autophagic flux using the HiBiT-LC3 reporter was performed as in (Will et al., 2022). Stable LC3-HiBiT cells were seeded in 96-well white plates (and 96-well clear plates in parallel for assessment of the number of cells by Hoechst staining). After treatment luminescence was detected upon addition of Nano-Glo^®^ HiBiT Lysis containing LgBiT protein and substrate (Promega), according to the manufacturer’s instructions. Luminescence was detected in a plate reader (BioTek, Synergy H1) and normalized to the cell number.

### Nuclear Fractionation

Nuclear fractionation of cells was performed according to Peña-Llopis and colleagues (2011). Briefly, cells were seeded in 10 cm plates and the following day were treated with the corresponding treatment for 24 h. Cells were washed in cold PBS, scraped, and pelleted at 2,500 *g* for 5 min at 4C. Pelleted cells were resuspended in two volumes of hypotonic lysis buffer (10 mM Tris-HCl pH 7.4, 10 mM NaCl, 10 mM MgCl_2_) containing protease and phosphatase inhibitors (Roche) and incubated on ice for 10 min. NP40 was added to reach 0.1% and incubated for 10 additional minutes on ice. Nuclei were pelleted upon centrifugation at 2,500 *g* for 5 min at 4°C, and supernatant (cytosolic fraction) was collected into a fresh tube. Nuclei were washed in 0.1% hypotonic lysis buffer, resuspended in the same buffer and both nuclei and cytosolic fractions were lysed in lysis buffer (Vega-Rubin-de-Celis et al., 2010) for 10 min at 4°C. Equivalent amounts of the fractionated samples were analyzed by Western blot.

### Autophagy Western-Blotting

Western-blot analysis of autophagy proteins was performed as in (Vega-Rubin-de-Celis et al., 2018). Cells were washed in ice-cold PBS and lysed in lysis buffer (50 mM Tris-HCl pH7.4, 250 mM NaCl, 0,5% NP40 (Vega-Rubin-de-Celis et al., 2010)) containing protease and phosphatase inhibitors (Roche) for 10 min at 4°C. Cell debris was cleared by centrifugation at 16,000 *g* 10 min at 4°C. Protein concentration was assessed by Bradford assay, and samples were diluted in 4x Laemmli Loading Buffer, boiled and loaded in 12% SDS-PAGE gels. Proteins were transferred to PVDF membranes, blocked in 5% milk-TBST (10 mM Tris-HCl, 15 mM NaCl, containing 0.1% Tween-20) for 1 h at room temperature and incubated with the corresponding primary antibody (see Table 2) diluted in 3% BSA-TBST overnight at four°C. Membranes were washed in TBST and incubated in HRP-conjugated secondary antibody for 45 min at room temperature. Membranes were washed in TBST and developed using a ECL system (Amersham).

### GFP-LC3 Puncta Formation Assay

Cells were seeded in 24-well black imaging plates (Eppendorf) and transfected with a pBabe-GFP-LC3 plasmid reporter the next day. One day after transfection cells were treated with the indicated treatments for 24 h and harvested by fixation with 4% PFA in PBS for 10 min at room temperature. GFP-LC3 puncta per cell were assessed using a Zeiss AxioCam MRm microscope (Carl Zeiss).

### qRT-PCR

RNA extraction was performed using a Qiagen RNeasy kits, according to manufacturer´s instructions, and cDNA was synthesized from 1 μg of total RNA in all samples with a QuantiTect Reverse Transcription kit (Qiagen). qRT-PCR was performed as in (Mergener et al., 2021), using primers published elsewhere (Peña-Llopis et al., 2011).

## Results

### Nintedanib Does Not Inhibit SRC Activation in MPM Cell Lines

**FIGURE 1:**
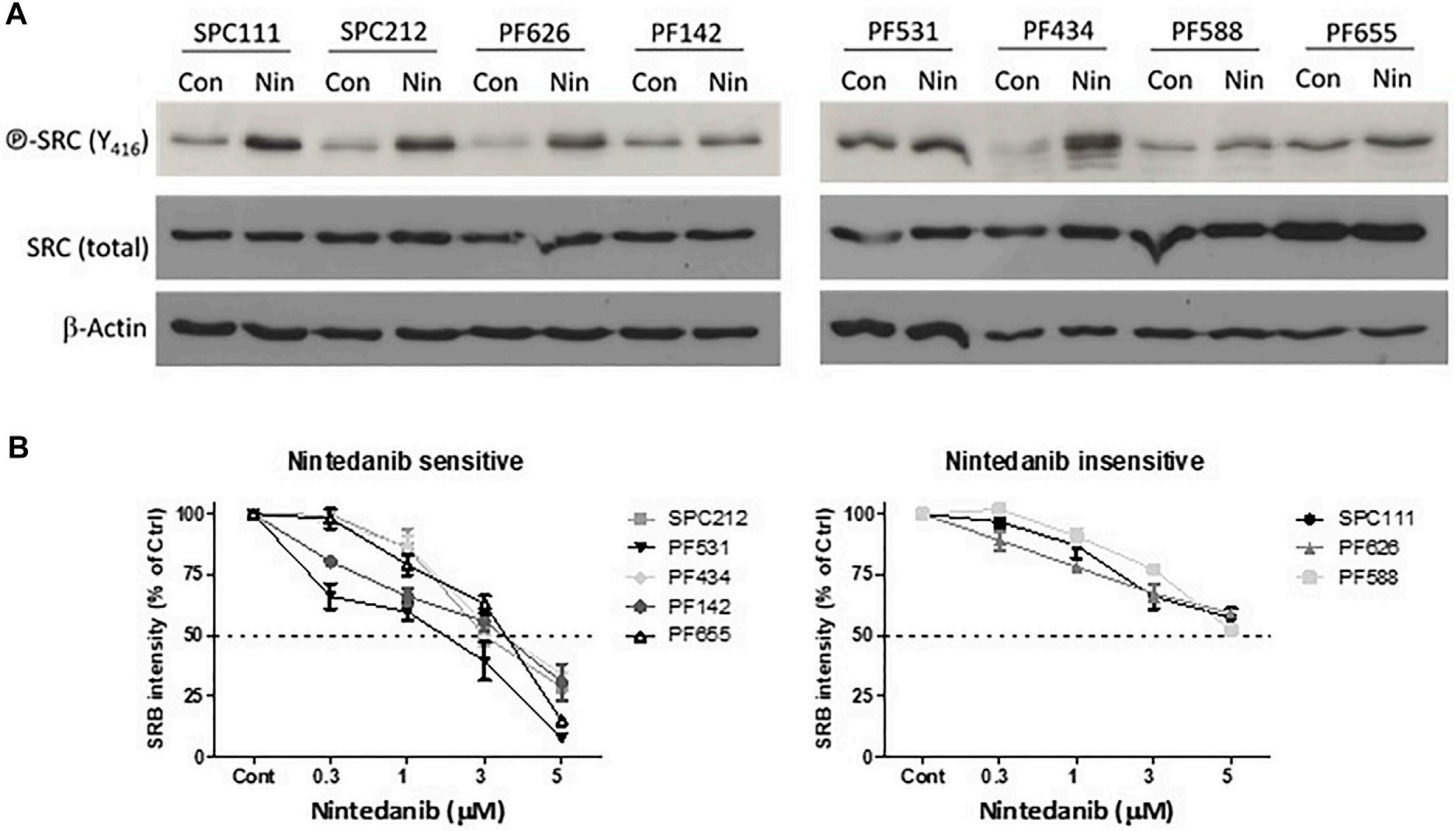
Nintedanib does not inhibit SRC in MPM cell lines **(A)** Cells were treated with 1 μM nintedanib for 24 h and protein lysates were analyzed by western blot **(B)** Cells were treated with nintedanib for 72 h. Cell viability was analyzed with SRB assay. All experiments were performed in triplicates. Bars represent means ± SEM from three independent experiments.

It was previously shown that nintedanib can inhibit the activity of the SRC kinase as assessed by kinase activity assays in myofibroblasts of the lung (Hilberg et al., 2008; Li et al., 2020). In order to investigate if it has a similar effect in mesothelioma cells we treated eight MPM cell lines with nintedanib and analyzed the activation of SRC (assessed by the phosphorylation of Y416). Surprisingly, we found that nintedanib did not reduce SRC phosphorylation (Y416) in any of the cell lines tested and in five of them SRC activation increased after treatment (Figure 1A). We determined the nintedanib sensitivity of all 8 cell lines and found that in five of them (SPC212, PF531, PF434, PF142, PF655) nintedanib substantially reduced the cell viability while 3 cell lines (SPC111, PF626, PF588) were insensitive (Figure 1B). Interestingly, we found that increased SRC phosphorylation (assessed by the phosphorylation of Y416) did not correlate with nintedanib sensitivity.

### The Effect of Dasatinib and Nintedanib Combined Treatment in Cell Cycle and Survival Is Cell Line-dependent

**FIGURE 2:**
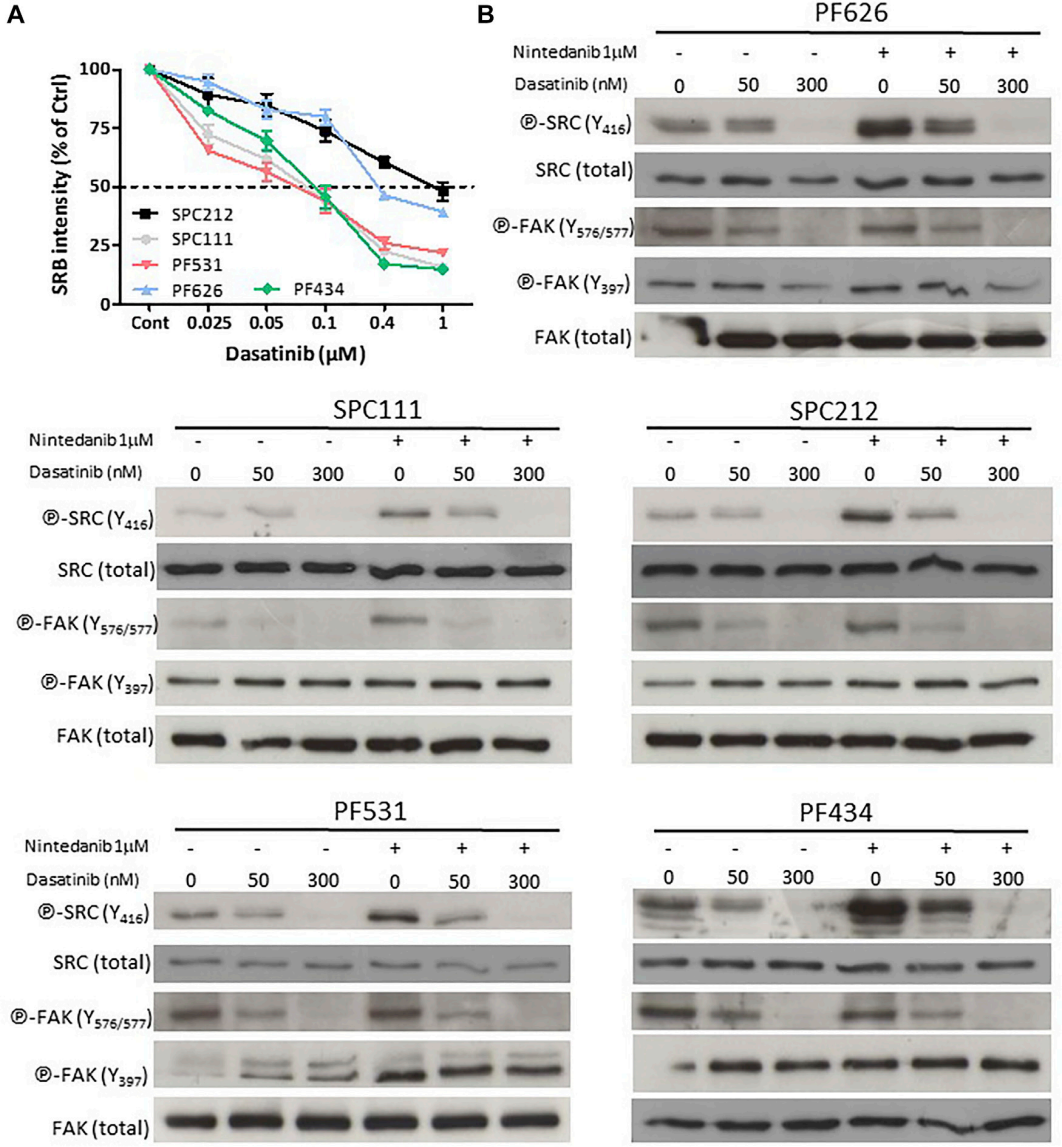
Dasatinib reversed the nintedanib-induced SRC activation **(A)** Cells were treated with dasatinib for 72 h and cell viability was analyzed with SRB assay **(B)** Cells were treated with 50 nM or 300 nM dasatinib, alone or in combination with 1 μM nintedanib for 24 h, and protein samples were analyzed by western blot. All experiments were performed in triplicates. Bars represent means ± SEM from three independent experiments.

Next, we analyzed if the SRC inhibitor dasatinib could revert the activation of SRC (assessed by the phosphorylation status of residue Y416) upon combination treatment with nintedanib. First, we determined the dasatinib sensitivity of the 5 cell lines where nintedanib treatment increased the SRC phosphorylation. We found that 3 cell lines (SPC111, PF531, PF434) showed high sensitivity to dasatinib while SPC212 and PF626 cells were less sensitive (Figure 2A). Then we measured the effect of dasatinib and nintedanib treatment (alone or in combination) on the activation of SRC and one of its targets, the Focal adhesion kinase (FAK) protein (as assessed by its phosphorylation status at Y576/577 and Y397) (Figure 2B). We found that in all cell lines dasatinib reversed the nintedanib induced SRC activation in the combination treatment. Dasatinib alone reduced SRC activation in all cell lines in a concentration-dependent manner and subsequently decreased FAK phosphorylation at Y576/577. Y397 residue is the auto-phosphorylation site of FAK. Its phosphorylation is initiated by integrins and growth factors and it enables the binding of SRC (Mitra et al., 2005). We found that the treatments did not decrease the phosphorylation of FAK at this site or even slightly increased it.

**FIGURE 3:**
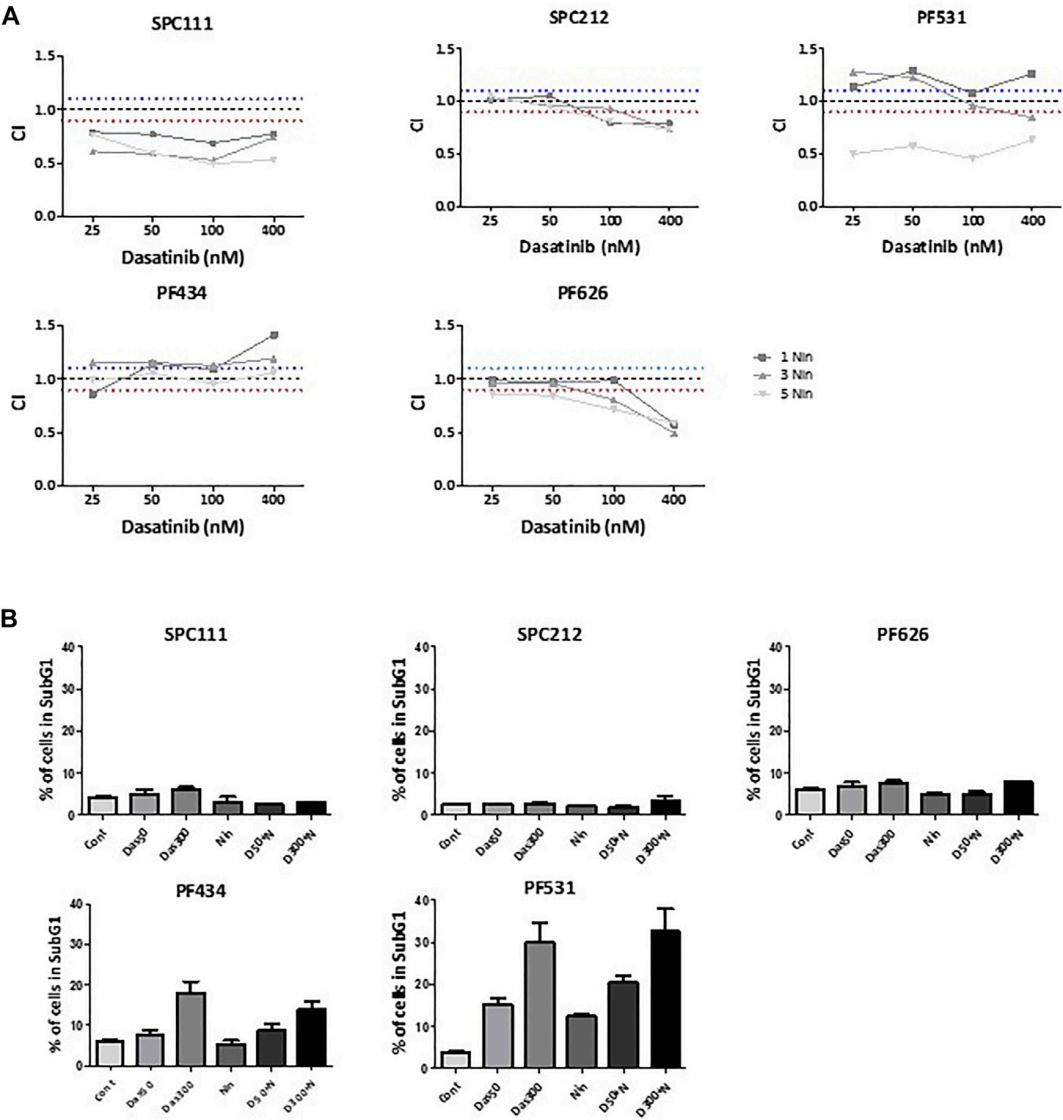
The effect of combination treatment of dasatinib and nintedanib on cell viability and cell cycle **(A)** Cells were treated with 1 μM, 3 μM, 5 μM nintedanib and 25 nM, 50 nM, 0.1 μM, 0.4 μM dasatinib in all combinations for 72 h and cell viability was analyzed with SRB assay. Combination index (CI) was calculated for each combination and that indicated synergism (CI < 0.9), additive effect (CI is between 0.9 and 1.1) or antagonism (CI > 1.1). Dotted lines indicate these cut-offs **(B)** Cell cycle analysis was performed after 50 nM or 300 nM dasatinib or 1 μM nintedanib treatment alone or in combinations for 72 h and the percentage of the cells in the subG1 phase was analyzed. Bars represent means ± SEM from three independent experiments.

Then we determined the effect of the dasatinib and nintedanib combination treatment on cell viability (Figure 3A, Supplementary Figure S1). We found that in SPC111 cells the combination treatment had a strong synergistic effect at all concentrations. In the case of the SPC212 and the PF626 cell lines, the effect was additive at lower treatment concentrations and at higher concentrations synergistic. In contrast, in the PF434 and PF531 cell lines there was an additive effect only at the higher treatment concentrations while at lower concentrations the interaction was antagonistic. To further analyze the effect of the combined treatment we performed cell cycle analysis (Figure 3B). Interestingly, we found that in PF531 cells both drug treatments strongly induced apoptosis even as single treatments. Similarly, in PF434 cells dasatinib increased cell death in a concentration-dependent manner. In the other 3 cell lines none of the treatment induced cell death and, while in SPC212 cells nintedanib treatment decreased the ratio of the cells in the S and the G2M phases, there was no alteration by any of the treatments of the cell cycle pattern of SPC111 and PF626 cells (Supplementary Figure S2). Interestingly, the synergistic effect of the combination treatment was most pronounced in these 2 cell lines. Since autophagy can also lead to reduced cell growth, and both drugs were described to initiate autophagy in other cell types, we investigated the effect of nintedanib and dasatinib treatment on autophagy in these cell lines.

### MPM Cells Differentially Modulate Autophagy in Response to Nintedanib and Dasatinib Treatments

**FIGURE 4:**
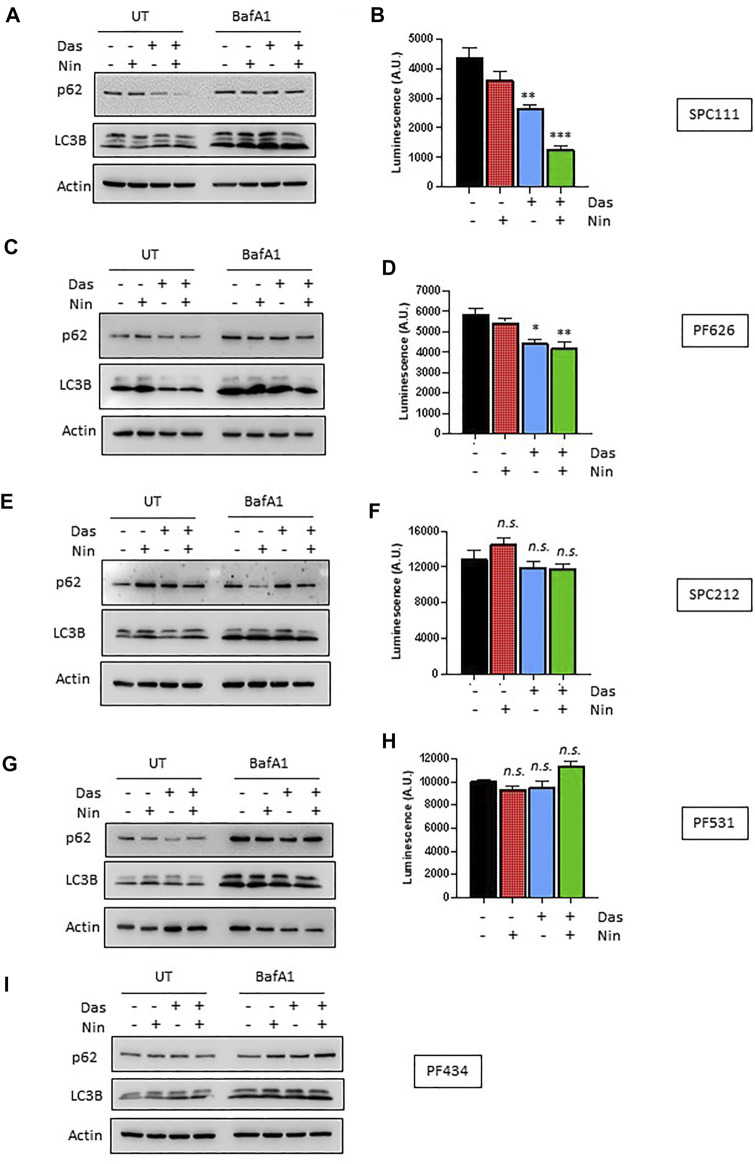
Autophagic response to dasatinib and nintedanib differs across different MPM cell lines. Autophagic flux of the indicated MPM cell lines was analyzed after treatment (Das: dasatinib 50 nM; Nin: nintedanib 1 μM; 24 h) with or without Bafilomycin A1 (Baf A1, 100 nM) by western-blot **(A,C,E,G,I)**, and HiBiT-LC3 luminescence **(B,D,F,H)**. Bars are average ±SEM of three independent experiments. *, *p* < 0.05; **, *p* < 0.01; ***, *p* < 0.001, *n. s.*, non-significant, *t*-test. Note that PF434 cells did not express the LC3-HiBiT after G418 selection despite multiple attempts.

**FIGURE 5:**
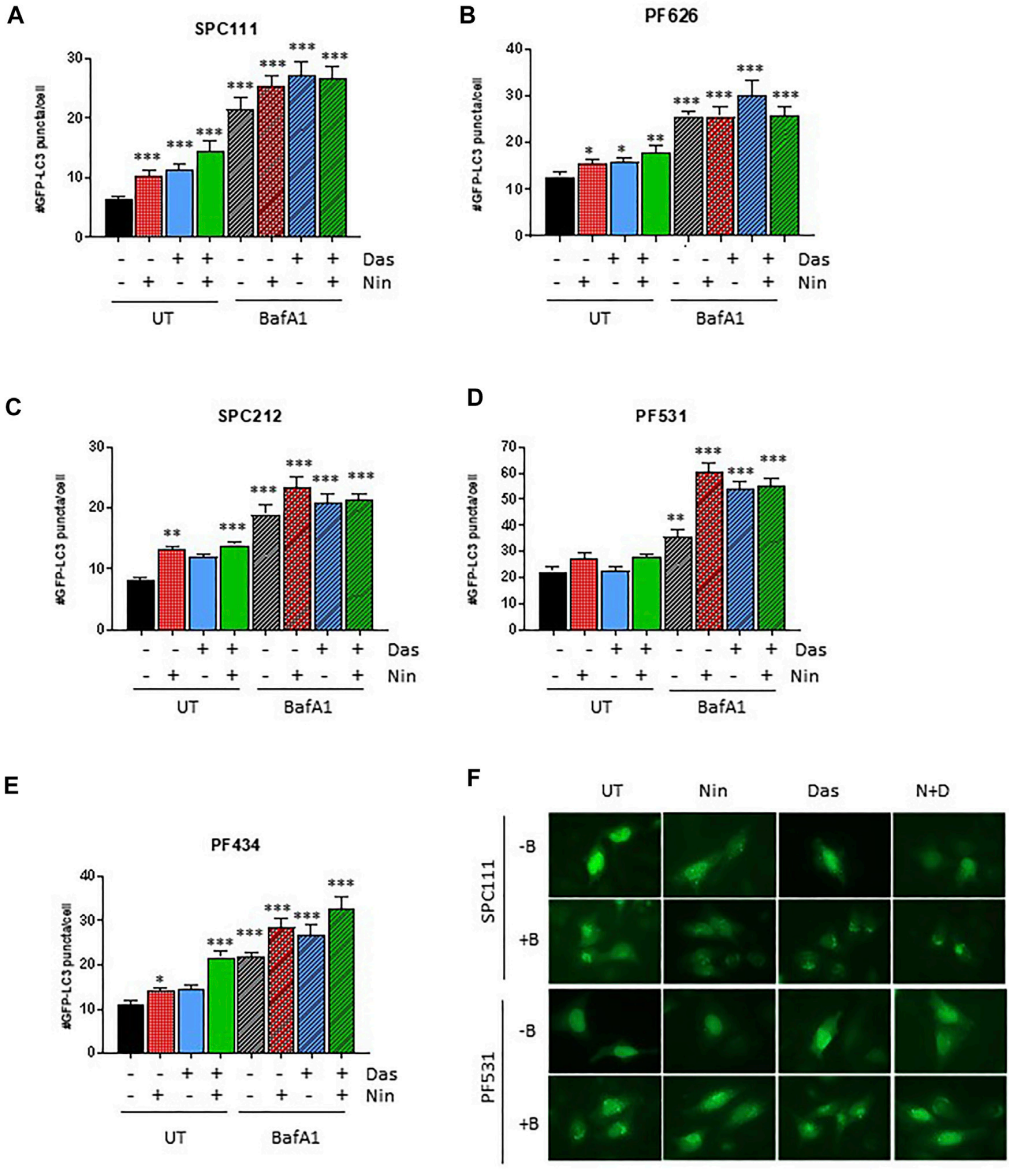
Autophagosome formation in response to dasatinib and nintedanib is dependent on the cell line **(A–E)** GFP-LC3 puncta formation was quantified after treatment (Das: dasatinib 50 nM; Nin: nintedanib 1 μM; 24 h; N + D, combination of nintedanib and dasatinib) with or without Bafilomycin A1 (Baf A1, 100 nM). Representative pictures are shown in **(F)**. Bars are average ±SEM of three independent experiments. *, *p* < 0.05; **, *p* < 0.01; ***, *p* < 0.001, *n. s.*, non-significant, *t*-test.

Nintedanib and dasatinib were shown to modulate autophagy in lung fibroblasts and in certain tumor cell lines, respectively. Accordingly, we analyzed the autophagic flux on the MPM cell lines panel (Figures 4, 5) by 1) western blotting of the autophagy marker p62 or the lipidation status of LC3B, 2) luminescence of the HiBiT-LC3 reporter, and 3) numbers of the autophagosome marker GFP-LC3. Interestingly, in the cell lines insensitive to nintedanib (SPC111 and PF626), autophagy was significantly induced upon treatment as assessed by degradation of the autophagy substrate p62 and increased abundance of the LC3B autophagosome-associated lipidated, fast moving LC3B-II form (Figure 4A,C), decreased readings of the HiBiT-LC3 reporter (Figure 4B,D), and increase on the number of GFP-LC3 puncta (autophagosomes) (Figure 5A,B) In addition, treatment with Bafilomycin A1 (an inhibitor of vacuolar H + -ATPases), induced further accumulation of GFP-LC3 puncta, as well as p62 and LC3B, suggesting that the observed changes are due to an increase in the autophagic flux rather that the blocking of the autophagic maturation. On the other hand, in SPC212, PF434 and PF531 cell lines, that are more sensitive to nintedanib, no significant autophagy induction was detected by the same assays. Taken together these data suggest that some MPM cell lines induce autophagy as a protective mechanism of resistance against nintedanib and dasatinib treatments.

### Nintedanib and Dasatinib Treatments Induce TFEB Nuclear Translocation Independently of mTORC1

**FIGURE 6:**
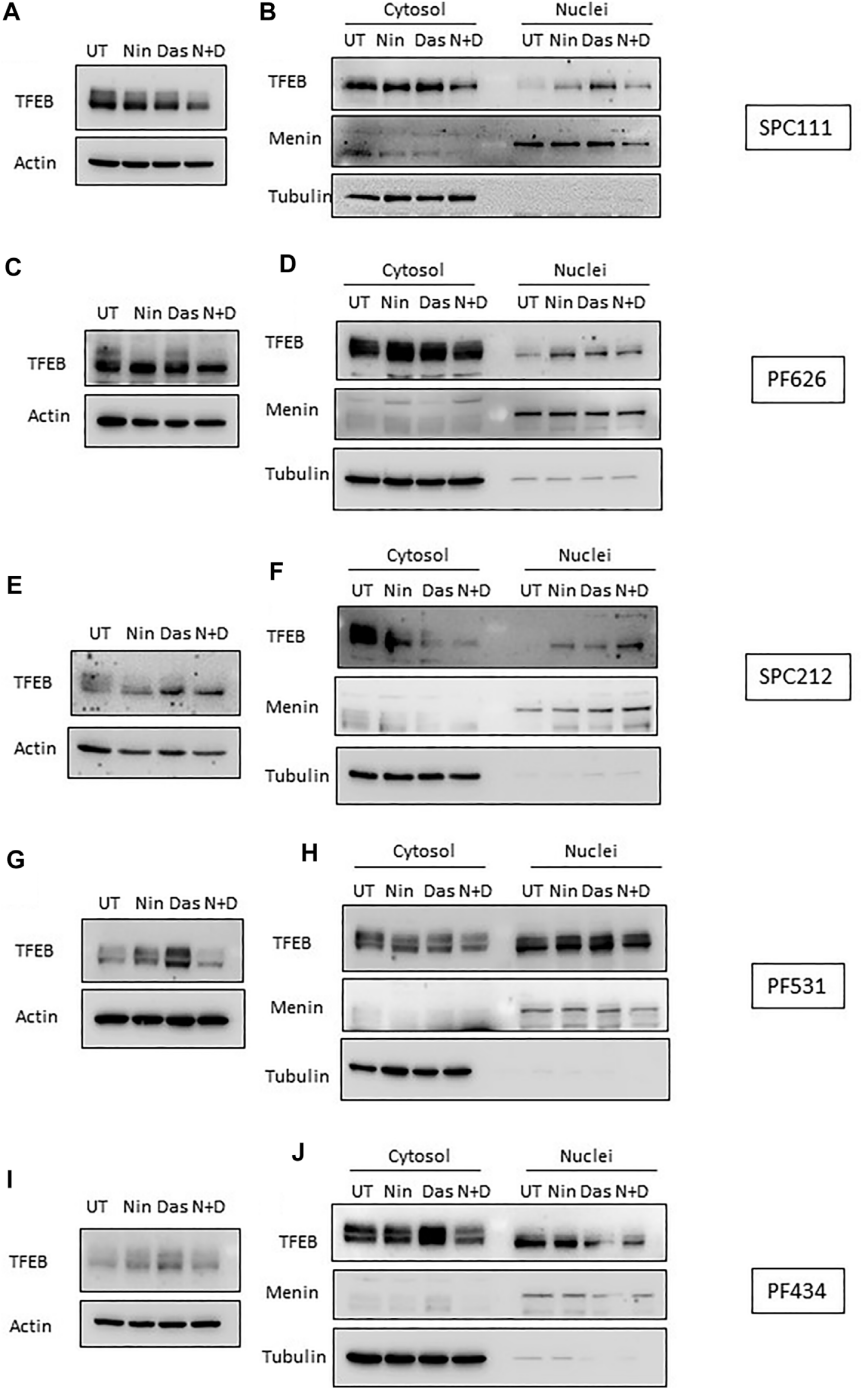
TFEB is de-phosphorylated and translocates to the nucleus upon dasatinib and nintedanib treatments. TFEB mobility shifting was analyzed by western-blot **(A,C,E,G,I)**, and its localization was determined by nuclear fractionation **(B,D,F,H,J)** upon treatment with the indicated treatments (UT, untreated DMSO control; Das: dasatinib 50 nM; Nin: nintedanib 1 μM; 24 h; N + D, combination of nintedanib and dasatinib). Menin and Tubulin were used as markers for purity of the nuclear and cytosolic fractions, respectively.

**FIGURE 7:**
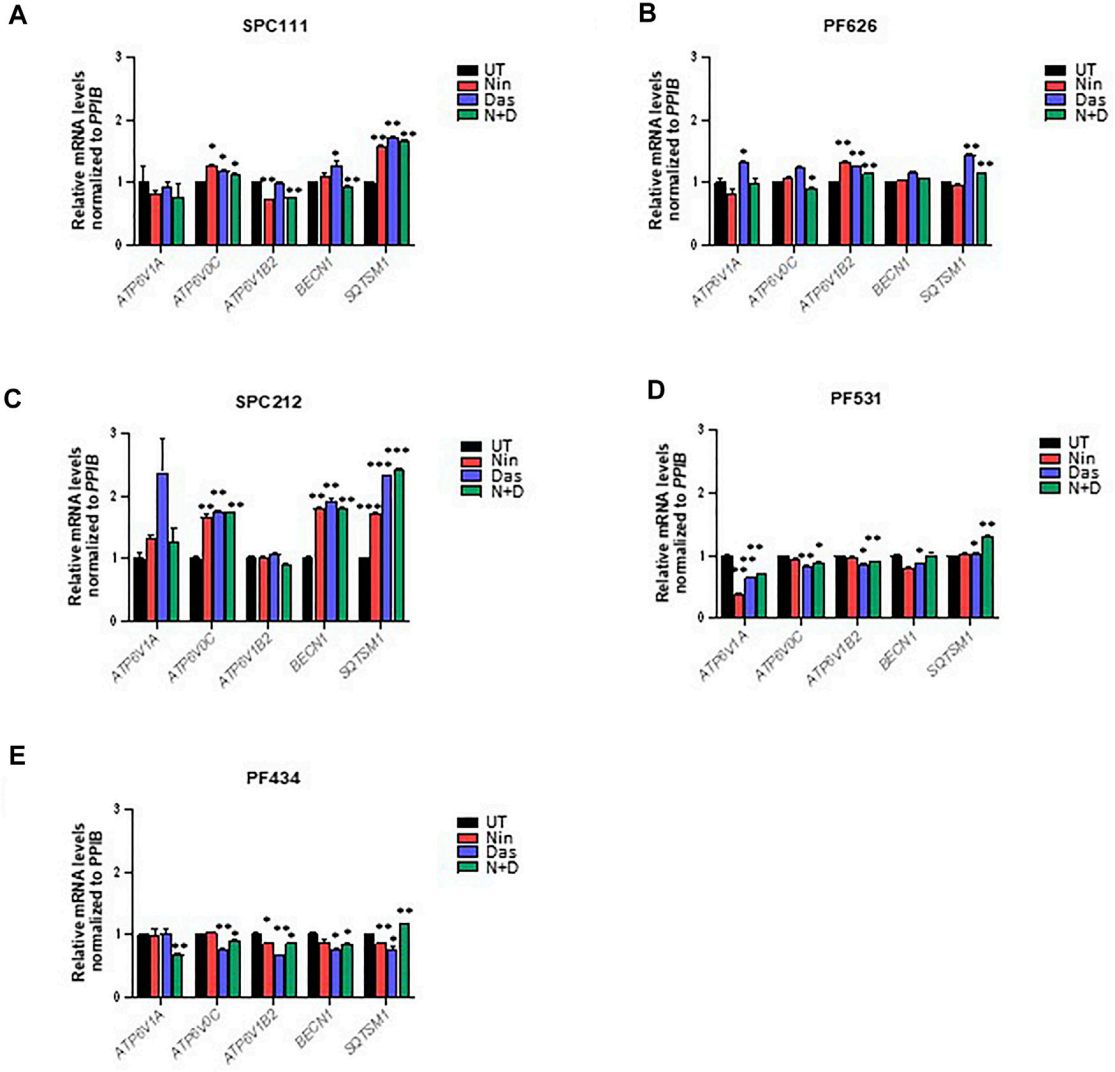
TFEB target genes are only induced in some MPM cell lines upon dasatinib and nintedanib treatments. Expression of the indicated TFEB target genes was analyzed by qRT-PCR after treatment for 24 h of 50 nM dasatinib and 1 μM nintedanib in **(A)** SPC111, **(B)** PF626, **(C)** SPC212, **(D)** PF531, and **(E)** PF434 cell lines. UT, untreated (DMSO) control; Nin, nintedanib; Das, dasatinib; N + D, combination of nintedanib and dasatinib. Bars are average ±SEM. *, *p* < 0.05; **, *p* < 0.01; ***, *p* < 0.001, *t*-test.

TFEB is heavily phosphorylated and modifications in its phosphorylation status can be observed through mobility shifting in western blot (Peña-Llopis et al., 2011). Thus, we assessed whether treatment with nintedanib or dasatinib (or in combination) altered the phosphorylation pattern of TFEB. Our results indicate that in all MPM cells treated with nintedanib and to a lesser extent, with dasatinib (alone or in combination) induced an accumulation of faster migrating forms of TFEB, suggesting an increased de-phosphorylation (Figure 6A,C,E,G,I). Such mobility shifting correlated with a clear accumulation of nuclear TFEB as determined by biochemical fractionation followed by western-blot in SPC111, SPC212 and PF626 (Figure 6B,D,F). In the PF531 and PF434 larger amounts of nuclear TFEB was present already in basal conditions (Figure 6H,J). Increased in nuclear localization of TFEB is typically associated with an increased transcription of multiple target genes, including lysosomal genes (*vATP6V0C, vATP6V1A, CREG*) as well as autophagy genes (such as *ATG4, BECN1, UVRAG*). Thus, we analyzed the expression of some of TFEB target genes in all cell lines after treatment with nintedanib and/or dasatinib (Figure 7). Surprisingly, the response was very heterogeneous across the different cell lines, and most of them do not strongly upregulate the lysosomal or autophagic genes analyzed. Only SPC212 cells responded to the treatments by a solid induction of *vATP6V0C, BECN1 and SQTSM1*. Therefore, TFEB does not seem to respond to nintenadib and dasatinib by inducing a transcriptional lysosomal or autophagic response although it is translocated to the nucleus across most cell lines.

**FIGURE 8:**
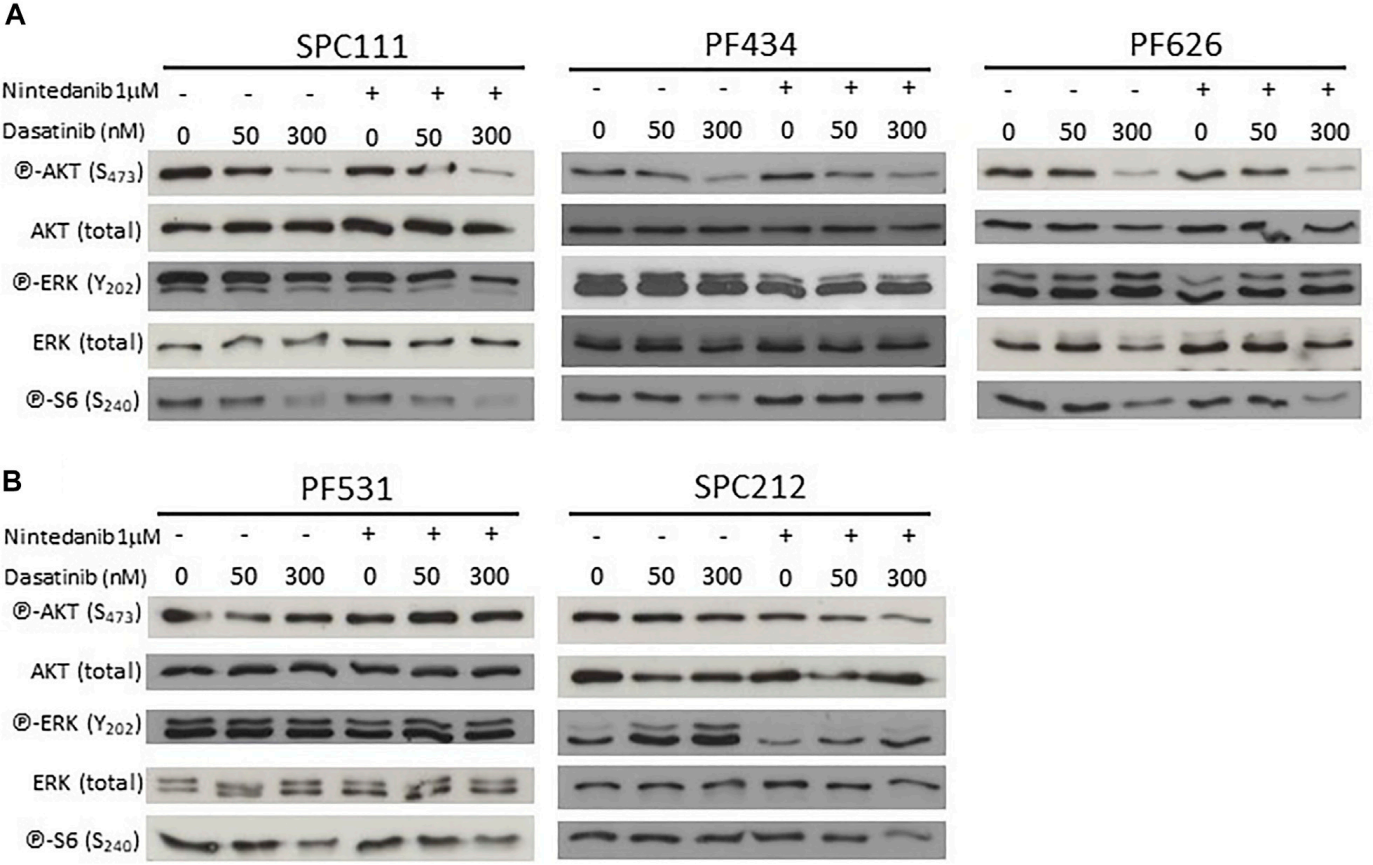
Nintedanib treatment does not affect the AKT-mTORC1 or the ERK pathways while dasatinib decreases AKT and pS6 activation in SPC111, PF434 and PF626 cell lines **(A)** but not in PF531 and SPC212 cells **(B)**.

TFEB phosphorylation and localization is regulated by multiple kinases, including mTORC1 (Peña-Llopis et al., 2011; Martina et al., 2012; Roczniak-Ferguson et al., 2012; Settembre et al., 2012; Vega-Rubin-de-Celis et al., 2017), AKT1 (Palmieri et al., 2017) and ERK (Settembre et al., 2011). Accordingly, we analyzed the phosphorylation status of AKT1 and ERK, as well as the mTORC1 activity through its downstream target S6. We found that dasatinib treatment reduced AKT1 phosphorylation in SPC111 and PF434 cells in a concentration dependent manner while in PF626 cells only at the higher concentration (Figure 8A). S6 phosphorylation was decreased only by high concentrations of dasatinib (300 nM) in these cell lines. In contrast, in SPC212 and PF531 cells none of the treatments affected AKT1 or S6 phosphorylation (Figure 8B) suggesting that the effects observed in TFEB localization are independent of mTORC1. In addition, ERK activation was only altered by the treatments in the SPC212 cells, where nintedanib treatment decreased ERK activation while dasatinib slightly increased it. Taken together, these data indicate that TFEB nuclear translocation upon dasatinib and/or nintedanib treatment is independent of the AKT1-mTORC1 and ERK1/2 pathways.

### Combination Treatment With Nintedanib and Autophagy Inhibitor 3-MA has a Synergistic Effect in SPC111 Cells

**FIGURE 9:**
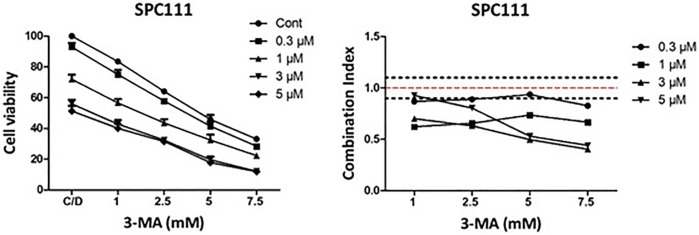
Combination treatment with nintedanib and autophagy inhibitor 3-MA has a synergistic effect in SPC111 cells. Cells were treated with 0.3, 1, 3, 5 μM nintedanib and 1 mM, 2.5 mM, 5 mM, 7.5 mM 3-MA in all combinations for 72 h and cell viability was analyzed with SRB assay. Combination index (CI) was calculated for each combination and that indicated synergism (CI < 0.9), additive effect (CI is between 0.9 and 1.1) or antagonism (CI > 1.1). Dotted lines indicate these cut-offs.

Since we have found that nintedanib induced autophagy more in the resistant cell lines we investigated if inhibition of autophagy in combination with nintedanib further decrease the viability of the cells. We found that combined treatment of nintedanib with autophagy inhibitor 3-MA had a strong synergistic effect (Figure 9). These results show that nintedanib induced autophagy has a cytoprotective effect in SPC111 cells.

## Discussion

Nintedanib (BIBF1120) is a small-molecule tyrosine kinase inhibitor (TKi) with a strong anti-angiogenic effect that could potentiate the effect of conventional chemotherapies. A promising phase II trial in mesothelioma patients showed that there are certain patients that could benefit from the addition of nintedanib. In order to identify these patients we need to find predictive biomarkers and to better understand potential resistance mechanisms. In *in vitro* kinase assays nintedanib inhibited the SRC kinase at pharmacologically relevant concentrations (IC50: 156 ± 40 nmol/L) (Hilberg et al., 2008). It was also demonstrated that nintedanib attenuates pulmonary fibrosis by reducing the activation in Wnt3a-induced myofibroblast through suppressing SRC kinase activation (Li et al., 2020). Furthermore, nintedanib decreased lung fibrogenesis and TGF-β1-induced epithelial-mesenchymal transition in mice after mechanical ventilation partially by inhibiting the SRC pathway (Grosso et al., 2017). It has been previously shown that SRC protein is strongly expressed in both MPM cell lines and tumor samples. Furthermore, the active, Y419 phosphorylated form of SRC was also present in cell lines and in high percentage of the tumors as well (Tsao et al., 2007). Similarly, we found that in all of the investigated eight mesothelioma cell lines SRC was strongly expressed and activated to a varying extent. However, nintedanib treatment did not decrease SRC activation in any of the MPM cell lines and in five of them we detected a paradoxical activation. Previously, it was shown that TKi can lead to SRC activation. In lung cancer cell lines insulin-like growth factor receptor (IGF-1R) inhibitor linsitinib treatment shortly decreased SRC activity but in a few hours SRC activity returned to an even higher level. However, combined treatment with linsitinib and dasatinib effectively lowered SRC activation for prolonged periods of time (Min et al., 2015). An inverse correlation between linsitinib sensitivity and phosphorylated SRC was also found. We determined the nintedanib sensitivity of our cell line cohort and found that 3 cell lines were highly sensitive (PF531, PF142, SPC212), two moderately sensitive (PF434, PF655) and three not sensitive. This is in good accordance with previous results that also showed that MPM cell lines strongly vary in their sensitivity to nintedanib (Laszlo et al., 2018). However, neither SRC base line phosphorylation, nor its further activation by nintedanib was dependent on the nintedanib sensitivity of the cells. To determine if combined nintedanib and dasatinib treatment can reverse the activation of SRC, first we analyzed the dasatinib sensitivity of the cells. We found that 3 cell lines were sensitive to dasatinib with an IC50 around 100 nM, while two were insensitive with an IC50 over 500 nM. This diversity in the sensitivity of MPM cell lines corroborated the results of previous studies (Tsao et al., 2007; Monica et al., 2016). Upon combination treatment dasatinib could reverse the effect of nintedadib on SRC activation already at the lower 50 nM concentration in all cell lines similarly to the results of the linsitinib and dasatinib combination (Min et al., 2015). We found that in 3 cell lines (SPC111, SPC212 and PF626), the combination treatment had an additive or synergistic effect and in these cells there was no cell death induction. While in 2 cell lines, there was an additive effect only at the higher treatment concentrations and in these cells dasatinib initiated cell death. Similarly, in other studies SRC inhibitors could induce cell death and cell cycle arrest in a subset of mesothelioma cell lines (Tsao et al., 2007; Indovina et al., 2012). It has been shown that TKIs can induce autophagy and this effect is independent from their target molecule. Tanaka et al. compared several TKI inhibitors, including dasatinib, and found that the autophagy inducing capacity of the inhibitors was cell line dependent and had a cytoprotective function (Tanaka et al., 2020). Since autophagy has been shown as a resistance mechanism in MPM cells, and both nintedanib and dasatinib were found to initiate autophagy in other cell types we analyzed if autophagy played a role in our new MPM cell panel, and especially in the drug resistant cell lines. Our autophagic flux analysis suggests that the cells more resistant to nintenadib induce a stronger autophagic response perhaps as a mechanism of defense against external insults. Although the autophagic response across the different cell lines is heterogeneous (perhaps also influenced by the large genetic diversity of the cell lines characteristic of MPM) there is a trend towards a stronger autophagic response upon treatment on the more resistant cells. This suggest that autophagy induction is a resistance mechanism in this context. Indeed, some of the more sensitive cells to both nintenadib and dasatinib (PF434 and PF531) do not induce autophagy upon treatment. However, in the more nintedanib resistant SPC111 cells combination treatment with autophagy inhibitor 3-MA had a synergistic effect. 3-MA inhibits the early steps of the autophagic process and was shown to increase the sensitivity of cancer cells to traditional chemo- and radiotherapy (Xiao et al., 2021). The correlation between tumorigenesis and autophagy is complex and context-dependent, and the first association between cancer and the autophagy machinery derived from observations in mice deficient for Beclin 1, since *Becn1* ^+/−^ mice spontaneously develop multiple malignancies at a high rate (Qu et al., 2003). Later on, increased tumorigenesis was also described in other *Atg*-deficient mouse models (Poillet-Perez and White, 2019). Such effects are probably due to the implications of autophagy in maintaining genomic stability and suppressing oxidative stress, suggesting a protective role of autophagy in early stages of tumor development. Autophagy also has a number of pro-tumorigenic functions that are believed to fuel already established tumors (Mizushima and Levine, 2020), and therefore inhibition of autophagy can be beneficial for cancer treatment, also because tumor cells rely more on autophagy for survival than normal cells. In many instances chloroquine (or hydroxychloroquine) are being investigated as inhibitor of the latter steps of the autophagy pathway, although the specificity of such inhibitors is limited (Manic et al., 2014), and clinical trials using these drugs showed a rather limited effect (Amaravadi et al., 2019; Mulcahy Levy and Thorburn, 2020).

The mechanism behind this differential response observed in our MPM cell lines remains to be determined, but using autophagy inhibitors may be an option to overcome these effects. Indeed, targeting autophagy vulnerabilities in MPM patients could be an option to improve the efficacy of the treatments.

Autophagy is also regulated at the transcriptional level by several transcription factors, including TFEB (Fullgrabe et al., 2016). TFEB is a master regulator of lysosomal biogenesis, and members of the MiT-TFE family of transcription factors are found in chromosomal translocations (maintaining the open reading frame but controlled by a stronger promoter) in several malignancies, characterized by an increased expression. Under normal, nutrient-rich conditions, TFEB is phosphorylated and bound to 14-3-3 proteins, thus retained at the cytosol. TFEB respond to multiple stress conditions by de-phosphorylation and translocation to the nucleus (Puertollano et al., 2018). A recent publication suggested that TFEB de-phosphorylation and nuclear translocation in a model of Niemann-Pick type C disease (NPC) upon c-Abl inhibition with dasatinib (Contreras et al., 2020). To our knowledge, our report is the first one describing a similar effect on MPM cells. In addition, we also report for the first time the modulation of TFEB localization by nintedanib, being both, the effects of dasatinib and nintedanib independent of mTORC1. A deeper understanding on the role of such nuclear translocation remains to be studied since its consequences on expression of the lysosomal and autophagy target genes is rather mild and very heterogeneous across different cell lines.

We found that autophagy induction by both dasatinib and nintedanib was independent of the AKT1-mTORC1-S6 pathways in MPM cells. Nintedanib treatment did not influence the activation of this pathway in any of the tested cell lines. The effect of dasatinib was dose and cell line dependent. Autophagy was induced after 50 nM dasatinib treatment. However, substantial decrease in the phosphorylation of AKT and S6 was only detected at much higher concentrations (300 nM). This was unexpected as in ovarian cancer cells dasatinib increased autophagy through a mechanism involving the decreased phosphorylation of AKT, mTOR, p70S6K, and S6 (Le et al., 2010). Similarly, dasatinib decreased AKT and S6 activation and increased autophagic cell death in glioma cells (Milano et al., 2009). Of note, dasatinib concentrations were over 100 nM in both of these studies. Although autophagy often facilitates drug resistance and limits therapy response, in certain cases, it can increase antitumor immune response after chemotherapy. Follo et al. have found that in 3D models of MPM tumors with low basal autophagy, upregulation of autophagy increased the release of damage-associated molecular pattern molecules after chemotherapy and initiated immunogenic cell death (Follo et al., 2019). It is important to note that nintedanib was applied in combination with chemotherapy in the referenced study.

Our study corroborates the finding that mesotheliomas are very heterogeneous and likely analysis of unique mutational and transcriptional changes will be required to identify adequate treatments. The three major histological subtypes of MPM have profound prognostic significance. The epitheloid subtype is the most common and confers the best prognosis while the rare sarcomatoid type has the worst outcome. The biphasic subtype contains cells with both characteristics. In our work, we used 2 cell lines with sarcomatoid (PF434, PF531), two with biphasic (SPC111, SPC212) and one with epithelioid origin. Importantly, we found that the two sarcomatoid cell lines were not only highly sensitive to both dasatinib and nintedanib treatments but in these cells the treatments induced cell death. After the successful phase II trial—that included 25% non-epithelioid cases - the failed extended phase III study was limited to MPM patients with epithelioid morphology. Our findings further support the notion that this change in the trial protocol might had an impact on the phase III study and highlights the need for further studies (Nowak et al., 2020).

## Data Availability

The original contributions presented in the study are included in the article/Supplementary Material, further inquiries can be directed to the corresponding author/s.
