# Supplementary material for: Nintedanib and Dasatinib Treatments Induce Protective Autophagy as a Potential Resistance Mechanism in MPM Cells
Source: Front Cell Dev Biol. 2022 Mar 22;10:852812. doi: 10.3389/fcell.2022.852812 (PMC8982261; doi:10.3389/fcell.2022.852812)
Supplement: Supplementary file 1 [file DataSheet1.PDF]

Supplementary Material

1 Supplementary Figures and Tables

1.1 Supplementary Figures

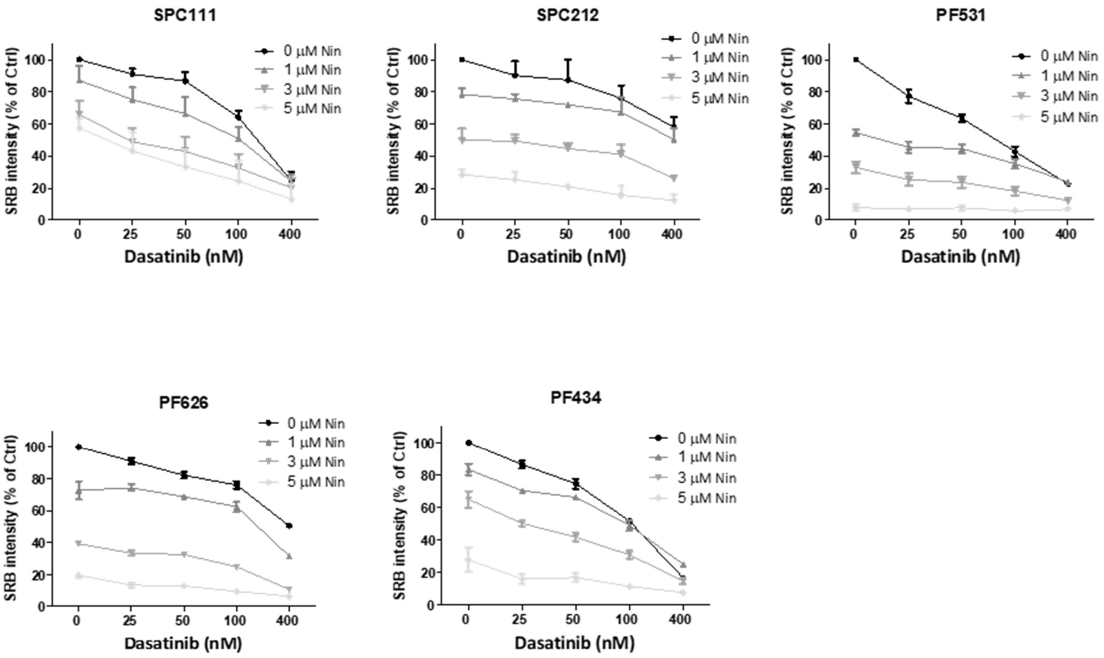

Supplementary Figure 1. The effect of combination treatment of dasatinib and nintedanib on cell viability. Cells were treated with 1 µM, 3 µM, 5 µM nintedanib and 25 nM, 50 nM, 0.1 µM, 0.4 µM dasatinib in all combinations for 72 hours.

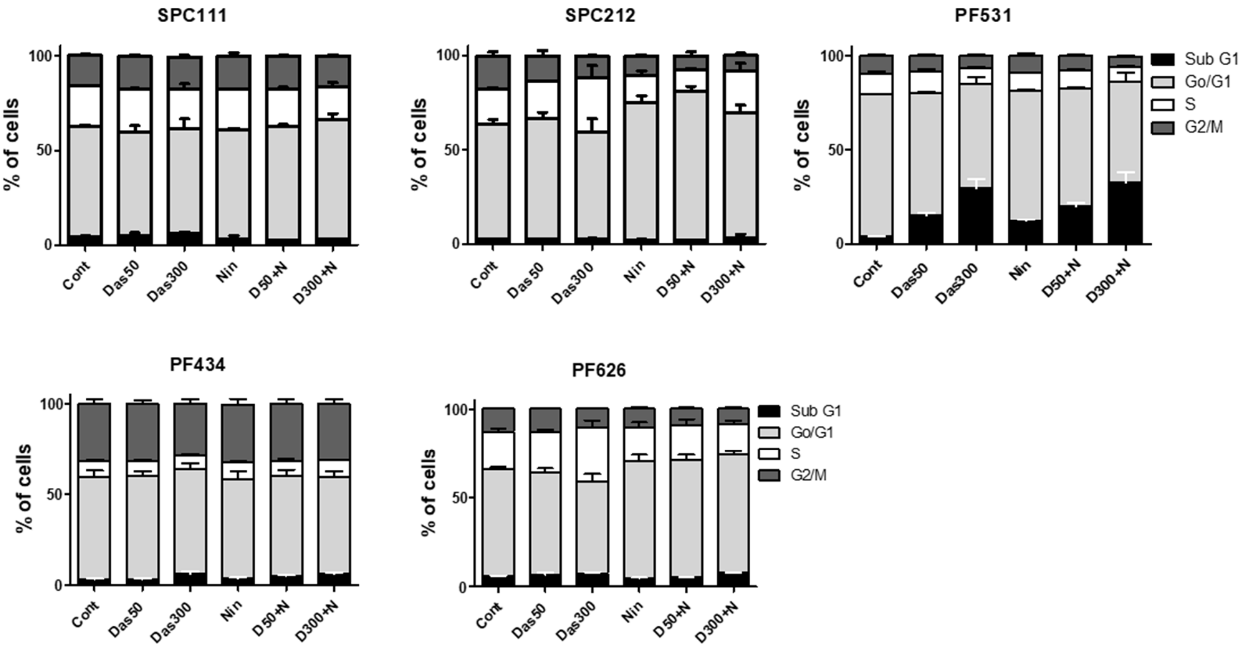

Supplementary Figure 2. Cell cycle analysis was performed after 50 nM or 300 nM dasatinib or 1 µM nintedanib treatment alone or in combinations for 72 hours. Bars represent means ± SEM from three independent experiments.

	IC_{50} values	
**Cell line \ Drug**	Nintedanib (µM)	Dasatinib (nM)
**SPC111**	5.9	86
**PF626**	4.4	458
**PF588**	4.2	196.8
**PF655**	3.3	10.6
**PF434**	2.9	108.6
**PF531**	0.9	94
**SPC212**	2.0	797
**PF142**	1.9	11.1

Supplementary Table 1. Drug sensitivity of mesothelioma cell lines. Cell lines sensitive to Nintedanib analyzed in this study are highlighted in blue, and the resistant cell lines in orange.
